# ’Joy, not sorrow’: Men’s perspectives on gender, violence, and cash transfers targeted to women in northern Ghana

**DOI:** 10.1016/j.ssmqr.2023.100275

**Published:** 2023-04-23

**Authors:** Audrey Pereira, Akalpa J. Akaligaung, Raymond Aborigo, Amber Peterman, Tia Palermo, Clare Barrington

**Affiliations:** a Department of Public Policy, University of North Carolina at Chapel Hill, USA; b Carolina Population Center, University of North Carolina at Chapel Hill, USA; c Navrongo Health Research Centre, Ghana; d Department of Epidemiology and Environmental Health, Division of Health Services Policy and Practice, University at Buffalo, USA; e Department of Health Behavior, Gillings School of Global Public Health, University of North Carolina at Chapel Hill, USA

**Keywords:** Ghana, Intimate partner violence, Cash transfer, Masculinities, Cash plus programming, Gender role strain

## Abstract

Evidence indicates that cash transfers can decrease intimate partner violence (IPV) against women, although most research has focused on women’s perspectives and experiences, with less attention to men. We analyzed data from four focus group discussions with male partners of women who participated in the Ghana Livelihood Empowerment Against Poverty (LEAP) 1000 cash transfer program. We elicited men’s perceptions of poverty, relationship dynamics, IPV and cash transfers targeted to their wives using thematic analysis. Men largely viewed the effects of the cash transfer as positive – they felt decreased provider role strain when women used the cash to cover household expenses such as food and school fees. Men also indicated that they felt respected when women used the cash to cover sudden expenses, such as funeral costs, thus preventing the need to borrow from community members and exposing their inability to fulfill provider roles. These feelings of relief and respect helped improve men’s overall wellbeing, their marital relationships and reduced the potential for IPV. Despite these positive results, men revealed that they still expected to be informed and consulted about the transfer and its expenditure, and felt disrespected when women did not do so, thus heightening the potential for household conflict. Further research and innovation in programming is needed to integrate gender transformative strategies into cash transfer programs, explicitly aimed at changing gender norms to enhance and sustain beneficial impacts on gender relations and IPV.

## 1. Introduction

Cash transfer programs are core policy instruments used by governments to reduce poverty and improve wellbeing among vulnerable populations (Beegle et al., 2018). A large body of evidence supports the effectiveness of cash across a broad range of economic, social and health outcomes, including positive outcomes for women and girls (Bastagli et al., 2016; Cirillo et al., 2021; Handa et al., 2022; Manley et al., 2022; McGuire et al., 2022; Palermo et al., 2019). In addition, cash provides participants with agency over use of funds, and increased dignity, particularly when given unconditionally, or without additional program participation requirements. Cash payments are often designed to target women directly, historically for *instrumental* rather than *intrinsic* reasons (Molyneux, 2006). For example, in many settings, women are primary caretakers of children, considered custodians of family food budgets, and perceived as having more time to dedicate to program activities. Thus, giving benefits to women is perceived to align with achieving program objectives related to food security, and children’s health and education.

Nonetheless, critics argue that cash transfers often fall short of being gender-transformative (Independent Evaluation Group, 2014; Peterman et al., 2020). A gender transformative approach requires that programming is purposefully designed to address gender inequities and the norms underpinning them (UNICEF, 2020). Doing so necessitates a relational approach that considers not only women’s (girl’s) views and outcomes in relation to the program but also those of men (boys). This requires a deeper understanding of intra-household dynamics, community norms and gender relationships, rather than solely focusing on programs designed to achieve impacts on women’s or girl’s outcomes.

Despite a broad body of literature on the impact of cash transfer programs on women in sub-Saharan Africa, few studies measure relational outcomes (Peterman et al., 2020). For example, studies may evaluate the impact of cash transfer programs on women and men’s labor force participation, but rarely analyze intra-household gender gaps in labor force participation, inequalities in earnings, or other gender equality outcomes. A primary example of this gap is assessing women’s experience of intimate partner violence (IPV), a pervasive global issue, underpinned by harmful hegemonic masculinities and norms that support male dominance over women (Sardinha et al., 2022). In Latin America and sub-Saharan Africa, for example, cash transfer programs have been shown to reduce physical, sexual, or psychological violence perpetrated by spouses or intimate partners (Baranov et al., 2020; Buller et al., 2018). However, most research on cash programming and IPV focuses on women and does not include men’s views and experiences. Men can provide key perspectives on impacts and understanding of why programming works, including mechanisms of impact, and how changes in design might bolster favorable impacts.

Evidence on mechanisms underlying protective impacts of cash transfer programmings on IPV reflect three main pathways: 1) improvements in economic security and emotional wellbeing; 2) reductions in intra-household conflict; and 3) increases in women’s empowerment (Buller et al., 2018). Following the framework laid out by Buller et al. (2018), there are at least two scenarios in which cash transfer programs could lead to an increased risk of IPV, both related to interactions within the household and men’s reactions to the program. First, following pathway 2, disputes between partners on how cash is spent may increase intra-household conflict. Second, following pathway 3, if women gain agency and power in the household as recipients of cash or complementary services, partners may act to counteract these shifts by reasserting their authority or attempting to extract the cash (Buller et al., 2018; Khoza et al., 2018). Therefore, a key question related to cash transfer programs that target women is: how do male partners of participating women react to their receipt of transfers in different settings?

Several qualitative studies have collected data from men to explore their views and reactions to their women partners receiving cash transfers. In an NGO-led conditional cash transfer targeting young women in South Africa, young male partners and peers were supportive of young women receiving the transfer but also expressed concern about losing authority in relationships (Khoza et al., 2018). Similarly, a mixed-methods study of Ethiopia’s Productive Safety Net Programme (primary a cash-for-work program) found that men had mixed reactions to the program, including both appreciation and fear of losing power in the household as a result of their wives’ empowerment (Ranganathan et al., 2022). In a mixed-methods evaluation of the Government of Zambia’s Child Grant Program, results showed that while women retained control over transfers, men’s perceptions of their role as primary decision-maker in the household remained unchanged (Bonilla et al., 2017). These findings highlight men’s mixed reactions, as well as the limits of cash transfer programs alone to achieve gender transformative impact in some settings.

The purpose of this study was to explore men’s reactions to their wives receiving cash transfers as part of the Livelihood Empowerment Against Poverty (LEAP) 1000 cash plus program in northern Ghana. The LEAP 1000 program is an unconditional cash transfer paid to eligible women in poor households, paired with a National Health Insurance Scheme (NHIS) premium waiver. In a quasi-experimental impact evaluation over a two year period, LEAP 1000 reduced the frequency of IPV (physical, emotional and combined IPV) among all women, as well as experience of 12-month IPV (physical, emotional and combined IPV) among non-polygamous women (Peterman et al., 2022). Supporting pathways for these impacts included reductions in household poverty and increases in women’s social support, economic standing, and health seeking behaviors. In a follow-up qualitative study with women in the LEAP program who experienced declines in IPV during the impact evaluation period, Barrington et al. (2021) found that all three pathways in the Buller et al. (2018) framework synergistically worked to reduce stress and tensions and increase women’s autonomy and confidence, leading to declines in IPV. However, the latter study suggested there was no evidence of fundamental transformative change in gender roles, even after four years of benefiting from LEAP 1000, raising questions about the sustainability of impacts beyond the program period.

To improve understanding of men’s perspectives on cash transfer programs and IPV, we analyzed data from focus group discussions (FGDs) with male partners of women participants of LEAP 1000 to assess two main research questions: 1) What are men’s perspectives of the relationships between poverty, wellbeing, and IPV? and 2) How do men perceive the impact of cash transfers on poverty, wellbeing and IPV?

## 2. Methods

### 2.1. The LEAP 1000 program

In 2008, the Ministry of Gender, Children and Social Protection (MoGCSP) launched LEAP to address extreme poverty and encourage long-term human capital development. It provides bimonthly unconditional cash payments ranging from 64 to 106 GH₵ to extremely poor households with orphans and vulnerable children, elderly with no productive capacity, and persons with severe disability. Eligibility was based on poverty status (and verified via proxy means test), as well as previously mentioned demographic criteria, identified via community targeting. In 2011, the MoGCSP and National Health Insurance Authority integrated an annual premium fee waiver into the LEAP program, including fees related to card processing, premiums, and renewals. In 2015, LEAP 1000 was piloted in 10 districts in northern Ghana adding a fourth eligibility criteria to include extremely poor families with pregnant women or infants under the age of one year. The overall objective of LEAP 1000 is to improve nutrition and development in the 1000 days from conception through 24 months, a key development window. By 2021, LEAP 1000 was integrated into the flagship national social protection program (LEAP), which is currently in all districts of Ghana, reaching more than 334,000 households.

### 2.2. Design and sample

We conducted four FGDs in two rural districts of northern Ghana: Garu-Tempane district in the Upper East region and Yendi district in the Northern region. These districts were chosen as they represented two distinct socio-economic environments present in the eight districts in the parent impact evaluation study. Although agriculture is the dominant livelihood activity in both districts, Garu-Tempane has 14 markets with substantial commercial and trading activities, whereas Yendi is more remote and rural, with only four markets. Both districts are generally patrilineal and traditionally male dominated (see Appendix for table of key development and contextual indicators by district). For example, women have less agency in decision-making as compared to men and are traditionally not allowed to own land regardless of the key role they play in agriculture production and processing. Fertility levels, which are associated with poverty, is higher in Garu-Tempane, as compared to Yendi. Polytheism is common within the districts with the populations being predominantly Muslim. Garu-Tempane has a lower proportion Muslim population as compared to Yendi (41 percent versus 67 percent). The proportion of women aged 15–49 years with one or more co-wives is also lower in Upper East region (including Garu-Tempane, at 26 percent), as compared to the Northern region (including Yendi, at 38 percent) (Ghana Statistical Service (GSS), Ghana Health Service (GHS), and ICF International, 2018). However, both these regions are higher than the overall national prevalence of polygyny, which is approximately 14 percent. In the LEAP 1000 impact evaluation data, 62 percent of LEAP 1000 eligible women reported experiencing emotional IPV, 38 percent physical IPV and 19 percent sexual IPV in the 12 months preceding the survey (Peterman et al., 2022). These prevalence levels are well above national averages, indicating women targeted by LEAP may be particularly vulnerable to IPV.

Data collection took place in November 2019, concurrently with in-depth interviews with female LEAP 1000 participants (Barrington et al., 2021). By November 2019, female LEAP 1000 recipients had been receiving cash transfer and health insurance premium waiver benefits for four years. To maintain confidentiality and protect participants, FGDs with male partners were conducted in different communities than the in-depth interviews. FGD participants were partners of LEAP 1000 participants in communities that had participated in the LEAP 1000 impact evaluation from 2015 to 2017. Male partners were recruited by LEAP community focal persons in each community. Eligibility was confirmed for each potential participant by the study team before consent and participation. The total number of male participants was 35 (range of 5–11 per group). All men worked in subsistence agriculture with only one doubling up as a community health volunteer. The average age was 45 years (range: 25 to 75), with only seven out of the 35 men having any formal education.

FGDs followed a semi-structured guide including six main topics: (1) community challenges and resources; (2) household challenges; (3) men’s and women’s responsibilities; (4) control over income; (5) how men and women show each other respect; (5) IPV and community intervention in cases of conflict; and (6) general impact of LEAP 1000 on households. Field guides were designed to ease participants into discussing their marital relationships and perceptions of IPV. FGDs were facilitated by a male moderator and a female observer who were Ghanaian researchers experienced in qualitative methods; some had worked on the LEAP 1000 evaluation. Moderators and observers were from the regions where the fieldwork was conducted and were fluent in the local languages. Observers took notes on key themes, group dynamics, and non-verbal communication. While starting with the questions on the guide, moderators probed for more depth on each theme and encouraged participants to comment and engage in dialogue. Recurring themes related to our main topics of interest were monitored, including perceptions of LEAP 1000 impact on IPV, household challenges, and men’s and women’s responsiblities. With four focus groups, we felt we had reached saturation of our main topics of interest along with identifying several new topics for further inquiry, which is consistent with recent sample size recommendations for focused studies with homogenous populations (Hennink & Kaiser, 2022). FGDs were conducted in Kusaal in Garu-Tempane and Dagbani in Yendi, audio recorded and transcribed directly into English by experienced transcriptionists. Authors AA and RA, who are fluent in English and the local languages, conducted quality control of the transcription and translation.

The study was approved by the Institutional Review Board of the University of North Carolina at Chapel Hill and that of the Navrongo Health Research Centre. Informed consent was obtained from all FGD participants who each received two bars of soap for their participation.

### 2.3. Analysis

Data analysis occurred concurrently with data collection, as the observers took detailed notes during the FGDs. The first author expanded the fieldnotes with information from the transcripts, adding additional detail and identifying salient quotes. The first author then developed a codebook including deductive codes based on the main topics covered in the guide and inductive codes based on emergent themes that came up in reviewing the data (Gibbs, 2022). These included gender role strain, or the stress that men feel related to their ability to meet their provider role (Pleck, 1987, 1995). While coding, the first and last authors regularly discussed, and the first author iteratively added and merged codes, returning to each transcript after every modification to ensure consistency. All coding was conducted using DeDoose software (Dedoose, 2015). After coding, we developed structured code summaries to systematically identify common themes across codes and FGDs. All authors were involved in interpreting and re-contextualizing through the process of writing the results. Our study guides and analysis was informed by the Buller et al. (2018) framework, alongside other more recent literature on gender and cash transfer programs.

## 3. Results

We first describe men’s perceptions of the relationship between poverty, gender, and IPV. We then examine men’s perspectives on the impact of LEAP 1000 on relieving men’s gender role strain and protecting men’s and household’s reputations in the community. Finally, we discuss how men viewed the impact of LEAP 1000 on women’s autonomy, relative bargaining power and IPV.

### 3.1. A context of poverty and gender role strain

“The only problem is the poverty – other than that, there is no challenge.” (40 years, Garu-Tempane)

Men identified poverty as the primary cause of household problems, shaping their emotional wellbeing, relationships with their wives, and ability to provide for their households. Men indicated that to adhere to their provider roles, they worked hard without rest to earn money and care for their families:

“The challenges for men are many. When your wife is sick it is up to you, if your child is sick, it is up to you. Children school fees is your responsibility. If you don’t work hard to earn money, you can’t take care of all these things. Every problem in the house is your responsibility as a man.” (42 years, Garu-Tempane)

Despite this burden of poverty, men reported shouldering most of the responsibilities in silence as expressed by a participant who said, “*So husbands have a load but they don’t carry it on their head*”, (Garu-Tempane). Participants considered the burden on men to be unequal compared to women, as noted in this exchange:

“P7: The man is the owner of the house, not the woman. All the work is on the man … First is the farming. We farm to get food to take care of the children. Secondly your wife’s parents’ home has their own problems that you have to attend to …. So, there is pressure on the husband. (67 years) I: Thank you. Number 9 do you have any response?

P9: I want to add on to what he just said. It is the same. All the struggles are to make sure you are able to pay school fees for the children. The woman is just an observer so if you don’t work hard you will not even have appetite to eat. So that is why we are struggling, it is not our fault. (45 years)” (Garu-Tempane)

This dialogue reflects the stress and frustration generated by men’s perceptions of being the sole providers in a context of constant demands.

These narratives around poverty interacted with household composition, with mixed responses regarding the experiences of men in polygamous marriages. One participant emphasized that poverty, rather than multiple wives, was the underlying problem for men, as reflected below:

“If you have more wives as a man you are blessed, the one with only one wife will suffer. But the poverty always brings the problems, but not the multiple wives. Two wives have a lot of advantages than one wife, but the poverty will bring the fight.” (40 years, Garu-Tempane)

Other men spoke about how having more than one wife helped the household. For example, one co-wife could help the husband on the farm while the other took care of household chores. Or if the household was in trouble and did not have money, one of the wives may have resources to share.

“I’ve one wife and if there is difficulty and my wife doesn’t have [money], it will be hard for us. However, with two-wife households, if one woman doesn’t have, another will have to support. But [in a] one wife-household, if you don’t have and your wife too doesn’t have, then you will be in trouble.” (45 years, Yendi)

Others however, reflected on the additional strain of having to provide equally for all wives and children. They noted that perceptions of favoritism could lead to conflict. One respondent provided an example of how his uncle was in such a situation:

“My uncle spoke about funerals at the wife’s parent’s home. It can happen that you have two or three wives and coincidentally, there are funerals in their homes at the same time. If you are able to attend one of the funerals with traditional drumming and dancing, your money has finished and so you can’t proceed to the other. The second wife will say you hate her. To solve the problem, you have to borrow. All these are challenges. The third wife parent’s funeral will also come and that is the challenge. You have to farm to sell to solve some of these problems.” (49 years, Garu-Tempane)

A clear and recurring theme was that poverty and the perceived inequitable burden on men created many challenges and negatively impacted their wellbeing. Participants reported feeling worry, sadness, stress, insomnia, and fear because of their inability to provide for their households. One man explained the link between his mental and physical health during challenging times:

“When it happens that way, you will think until you grow lean as if you are sick. Someone will wonder whether you are sick because you were okay but now slim. Your mind is not at rest and you can’t sleep at night like before. So, as you think and also not getting what you want, you will grow lean.” (49 years, Garu-Tempane)

and another spoke of the effects of poverty and consequent gender role strain on his emotional wellbeing:

“I: How do you, as heads of your household, feel when you are not able to meet your responsibilities?

P3: When that happens, you are always uncomfortable and irritable. Things you used to laugh over will now become serious matter” (45 years, Yendi)

Beyond such impacts on their own lives, men also discussed how the consequences of stress and their inability to provide for their households could affect other family members’ wellbeing. The following exchange demonstrates how the consequences of poverty, men’s inability to provide, and a lack of financial support can have long-lasting harmful effects on children and marital relationships:

“I: Has it ever happened that you do not have anyone in the household to [turn to for] support?

P4: Yes, when it happens that way that I do not have a turning point in the family, I can go to a neighbor and borrow and later pay back. (30 years)

I: When was the last time it happened to anyone?

P1: It has happened to me and it was my wife I asked and she supported me with money. But when one has no one to help that is where you will have sleepless nights. You will be worried and you may not be able to think right because you are the household head and responsible for taking care of needs of the household yet the resources are not there. (30 years)

I: How does that affect the household?

P2: It can lead to young girls following men just to get money and buy their needs. It can lead to teenage pregnancy and child marriage. (30 years)

I: What about you and your wives?

P1: The wives will not be happy with the household head and conflicts will start coming.” (Yendi)

Five men spoke of how young girls enter relationships or engage in transactional sex to obtain basic material needs and worries about teenage pregnancy:

“Your child needs pants and there is no money, she needs [a] uniform and there is no money. She will go somewhere for help, there is no empty help too, so the next thing is pregnancy yet we all trusted her. So is the poverty.” (75 years, Garu-Tempane)

Multiple participants also reported that they worried about their children falling sick and being unable to pay for medical costs:

“Sometimes our crops die as a result of our inability to get fertilizer for them. If you have a little money you plan to use for fertilizer, you might just end up using it to solve other problems that require urgent attention, like using it to take care of medical bills of sick children or family members.” (36 years, Garu-Tempane)

As noted in the above quote, men had to manage their limited resources across various household needs.

### 3.2. Poverty as the main cause of IPV

“Poverty can cause a man and his wife to fight.” (36 years, Garu-Tempane)

In addition to affecting their personal wellbeing and the wellbeing of their household, poverty-related gender role strain had the potential to trigger household conflict. One man clearly linked the peace in his household to his ability to provide:

“What I want to add is that if you were able to attend to your household needs, there will not have been a fight but since you aren’t, definitely there will be a fight”. (45 years, Yendi)

One participant indicated that when he felt stressed he was “*very quick tempered and easily irritable to things which under normal circumstances will not irritate [him]*” (45 years, Yendi). Men also stated their wives would become angry or upset with them if they could not meet their households’ needs, with the potential for tensions to escalate to violence, as evidenced in this exchange:

“P6: When that happens, anything you say that don’t merit insult – she will insult you …. (42 years)

I: What will follow after the insult?

P4: What will follow after the insult will be beatings. You will beat her and when you do she will go back to her [parents’] house leaving you.” (42 years, Yendi)

Even when men provided for their households, they stated that other factors, such as attempts to discuss management of household resources or women not fulfilling their responsibilities, could trigger conflict and household tensions. One participant spoke of food mismanagement in his household as an example of such a situation:

“After the farming season, whatever we get is supposed to be stored and used for our feeding [consumption]. But some women do not know how to manage food. They misuse the food and we run short before we farm again and there’s no way to get food again until it’s the next farming period. It’s not like we farm twice in a year. An attempt to show them how to manage the food could result in troubles at home. When the food finishes, the woman looks up to you and you have to provide food by all means because you married her, but that period is always a ‘wahala’ [suffering] period.” (26 years, Garu-Tempane)

This situation reflects the ingrained roles of men as providers and women as food managers; when such roles are questioned, conflict can result. Additionally, the expectation that men provide more food during the lean season could lead to further stress and the potential for IPV.

Men also cited women’s inappropriate behavior, insulting their husbands, or beating their children were sources of conflict. Two men blamed women more generally, simply stating: “*The women always have a way of looking for troubles*.” One respondent also suggested that peer influence caused misunderstandings in the home, leading to triggers for violence:

“It is as a result of peer influence. Most of it is from the women. If you have multiple wives, and you are conversing with one, the other will say you are talking about her and so will report to the friend. The friend will say don’t agree, respond back this way or that. If the woman is not sensible and takes all the bad advice from the friend, she will come and step on your toes, she will start insulting you. If you are quick tempered you will fight her back. But if the woman is wise she will know that the friend is interested in breaking the marriage. But the foolish one will come home and start fighting you.” (49 years, Garu-Tempane)

These narratives of blame for IPV related to women’s behaviors or peer influences point to underlying norms casting violence as an acceptable reaction to disagreements and conflict in the home.

### 3.3. LEAP 1000 impact: Gender role strain relief and protecting men’s reputation

“The support given to the women is helping them and us.” (40 years, Garu-Tempane)

The most direct impact of LEAP 1000 appreciated by men was relieving them of the burden of the household financial responsibilities that they struggled to fulfill. Men reported that women used LEAP 1000 cash transfers for household expenses, in particular nutritious food, school fees, and medical expenses, including renewing health insurance through the NHIS. Pregnant women also benefitted from being able to pay for medical costs.

“It has helped in some problems with regards to sudden issues, hospital bills and the rest. That is the first help. The second help is with regards to children not able to grow up well. Now they can buy good soup to prepare for the children to eat well. When they are also pregnant, they are able to do things to help the unborn child to grow well.” (37 years, Garu-Tempane)

The impact of the cash transfer was not limited to just covering expenses. Men reported that their wives also increased assets and investments, such as in poultry or small livestock, thus providing a safety net in the lean season or in case of shocks.

“The women who had a little something from it bought a small goat and hen to rear. The children nowadays easily get sick and when they do, the goats are sold to take care of their medical bills. So, I’d say the help was good.” (60 years, Garu-Tempane)

The extra cash to cover children’s needs and improve economic security through investments helped decrease men’s stress and improve their mental wellbeing. Two respondents emphasized the importance of the cash transfer in alleviating their “*wahala*” or suffering:

“The man is supposed to see to the wellbeing of the family but is sometimes not able to take care of everything because of the ‘wahala’ he is experiencing. The government, fortunately, is helping us with the ‘wahala’ through our women.” (26 years, Garu-Tempane)

The cash transfer also helped improve men’s self-esteem and identity as men.

“We used to sit in our houses with no goat or fowl to rear. Now, we are able to get at least a goat or hen to rear. Now, you can call yourself a man.” (62 years, Garu-Tempane)

This quote directly connects the economic impact of the transfer on men’s ability to protect their manhood, even though they are not the direct recipients of the cash transfer program. Men further reported that their wives’ contributions to household needs with LEAP 1000 cash helped protect their reputations, allowing them to maintain dignity and forgo embarrassment linked to poverty. The acquisition of assets was viewed as a benefit to the household,

“Now we are able to buy animals to rear and take care of basic needs. That’s how the money has helped the men. When somebody comes around and sees the animals, they’ll definitely say it’s yours and not the woman’s because you are the head of the household and that massages your ego as a man.” (26 years, Garu-Tempane)

Notable in this quote is the idea that livestock would be seen as belonging to the man even if it was procured using the cash transfer that was provided to the woman.

Men stated that their wives also used or lent them cash in times of need, such as paying for funeral costs, which prevented men from experiencing embarrassment. Men said they felt respected when their wives were able to step in and ease this gender role strain, which improved partner relationships.

“One evening a call came that my aunt is dead. So I first told my wife and she said they collected the money yesterday. I was able to pay for the funeral cost and everything without anybody noticing. If LEAP were not around, neighbors would have heard of my poverty situation. There was another day they told me the child was sick and was sent to hospital. Everything was paid. No one heard I was in need of money. If not because of LEAP, I would have been embarrassed, so it is good.” (75 years, Garu-Tempane)

This participant emphasized that he viewed his wife supporting their financial needs with LEAP 1000 cash as a sign of respect by protecting him from embarrassment. Of note, some men framed this financial help as borrowing and discussed that when they could not pay their wives back, they knew they could further count on their wives’ discretion to save them from embarrassment.

### 3.4. LEAP 1000 impact: Women’s financial autonomy and freedom from IPV

“That money is supposed to bring joy to the family and not troubles or quarrels … Joy, not sorrow”(36 years, Garu-Tempane)

When asked about whether the cash transfer brought misunderstandings or conflict, men overwhelmingly responded that it did not. As reflected below, participants viewed the cash as reducing conflict:

“[With] the help we are getting from the government, domestic fights have reduced because the little we get helps us to solve basic problems and that keeps the family going.” (36 years, Garu-Tempane)

Men stated that LEAP 1000 helped, rather than hurt spousal relationships, noting that they understood that the money was meant for the women, and they therefore, could not fight over it.

“That money should not bring any sorrow. I can’t fight over something that’s not mine. We should be thankful to the government for helping us and not fight over it. How can I fight over something that’s not mine?” (45 years, Garu-Tempane)

However, men expected their wives to inform them when they collected the money. All but one participant stated that their wives informed them about the cash transfer. The one man whose wife did not inform him felt excluded and disrespected, as reflected in the excerpt below:

“My wife takes it [the cash], but there is no respect between us. She never informs me after she has taken it. I never know the amount she takes whether its 10 pesewa or 20 pesewa, I don’t have an idea and I never ask.” (30 years, Yendi)

There was also no consensus on who ultimately retained control over the cash and approaches to decision-making ranged widely. Some men said that after taking care of children’s needs, women made decisions on spending the rest:

“When the women return with the money, they will show it to the husbands and if it is that the child has no books, or there is no soap, or whatever that is needed, she will use the money to buy those things. She will inform you the man what she is going to do, but it is the woman that spends the money.” (42 years, Garu-Tempane)

Others reported that men had more of a role in the decision-making process by providing suggestions or questioning women’s ability to spend or use the money prudently for the collective good of the household:

“The man and the woman decide what to do with the money. Even though the man’s name is not on the list of recipients, you can’t just leave the woman to decide what to do with the money because she is the direct recipient. If you leave the woman to decide for herself, she could use the money to drink and come home and insult you on top. So, you have to decide together what to do with the money.” (45 years, Garu-Tempane)

The nature of the expense also dictated the extent of control over the transfer. Men stated that women could take unilateral decisions if the expenditure involved an emergency:

“If the man goes to farm and leaves the wife and children in the house and something happens they take care of themselves before the man comes back from the farm.” (30 years, Yendi)

Overall, these diverging narratives reflected men’s perceived mixed opinions on LEAP 1000’s impact on women’s financial autonomy.

There were also contrasting opinions on whether the cash transfer had positive or negative effects on household members’ relationships. One man in a polygamous marriage spoke of how one wife becoming a participant put him under more pressure to help the other:

“[It’s] not all the women that benefit, those who don’t get will demand from you the man and if you are not able to get it for her, she thinks you don’t love her. Those who are benefiting cannot also share it like that with their colleague women [co-wives] at home. So, it’s a worry at home. It is as if the LEAP hated them and only love those who are benefiting.” (49 years, Garu Tempane)

However, others reported that participants helped their co-wives and the other children in the household. When asked about the relationship among co-wives one man expressed this sentiment:

“They are relating well with the other women at home. When they buy clothes, they show it to their colleague women [co-wives] at home. They buy soup to cook for everyone. They buy children clothes and their rivals [co-wives] thank them and the children are happy. It is sweet.” (67 years, Garu-Tempane)

## 4. Discussion

We examined men’s perceptions of gender roles, control over transfer money, and impact on IPV using four FGDs in northern Ghana. Men expressed that poverty constrained their ability to provide for their households, which they felt negatively affected their physical and mental wellbeing, marital relationships, and reputations in the community. Men were generally accepting of the cash transfer, and largely viewed its effects as positive. Wives used the cash to pay for household expenses, including children’s school fees, medical costs, and food during the lean season, which alleviated men’s stress related to their provider responsibilities. Furthermore, men provided examples of their wives covering sudden expenses, such as funeral costs, which protected their reputations. Men expect to be informed about and consulted regarding transfer money expenditure and felt disrespected in rare cases when women did not do so.

Men’s perspectives of poverty, gender role strain, and the impact of LEAP 1000 are similar to the narratives shared by women participants (Barrington et al., 2021). Both men and women’s narratives reflect the first pathway of the Buller et al. (2018) framework of improved economic security and wellbeing leading to reductions in IPV (Buller et al., 2018). Both men and women emphasized men’s inability to adhere to their provider roles as a trigger of IPV and viewed the cash as reducing men’s stress related to this and, consequently, reducing marital tension and IPV (Barrington et al., 2021). The cash transfer relieved men’s strain, both by reducing provider responsibilities and helping protect their reputations in the household and community. Men also commented on the subsequent positive impact of LEAP 1000 in preventing household conflict, the second pathway in the Buller et al. (2018) framework, as women’s increased access to cash for household expenses mitigated some of these tensions.

The impact of gender roles, norms, and consequent effects on men’s behavior, including IPV perpetration, have been documented in the African context. For example, a qualitative study with men and women in Tanzania revealed how women’s labor force entry and men’s loss of status as breadwinners could increase tension and potential for IPV in the household (Manji et al., 2020). Men were ridiculed for their inability to support their households and the gossip that their wives were having affairs. Consequently, men perpetrated IPV to reassert their status as head of household and with the hope of restoring their reputation with their peers. In our study, women lent cash from the transfer to their husbands in times of need, thus protecting men’s inability to provide from becoming known. In Rwanda, women’s work outside the home was accepted if men failed to fulfil their provider responsibilities, if women earned less and had lower status jobs than their husbands, and if women also fulfilled their domestic responsibilities (Stern et al., 2018). However, this work could be a source of conflict if men did not authorize the work, felt threatened, or if women neglected their domestic responsibilities, highlighting men’s authority. A study of the Government of Mali’s Jigisémèjiri cash transfer program found that cash given to male heads of household reduced men’s stress and anxiety, and women’s experience of emotional and physical IPV, and controlling behaviors in polygamous households (Heath et al., 2020). A companion qualitative study in Mali supported reduced gender role strain, improved emotional wellbeing, and decreased intrahousehold conflict pathways (Lees et al., 2021). Our findings add to this literature by highlighting that cash transfer programs targeted to women can promote reductions in men’s gender role strain through cooperation and sharing of resources within the household.

Men and women’s perspectives differed regarding men’s provider burden and their control over cash from the transfer. In the current study, men indicated that they felt the burden to provide for all household responsibilities and depicted a seemingly impossible pursuit of fulfilling this role in the context of northern Ghana. In contrast, women presented a more critical perspective saying “*men only give maize*”, depicting men as making more limited contributions to meet the basic needs of the household. Women emphasized the double burden that they felt to both provide for the household and protect men from feeling shame (Barrington et al., 2021). Even though men’s examples underscore how much women contribute to the economic wellbeing of the household, they clearly expected to be informed and consulted about how to spend the money received from the transfer. These results resonate with a mixed-methods study of the female-targeted Zambian Child Grant Program, which found that even though women were able to retain control over transfers and increased financial empowerment – entrenched gender norms dictating men as heads of households and as primary financial decision-makers remained unchanged (Bonilla et al., 2017).

Echoing findings from interviews with women, our findings also suggest that cash transfer programs alone may not automatically increase women’s agency or transform gender norms in the household or community. Cash and cash-plus programs that work within existing normative frameworks, where the man has control of any cash or asset inflow into the household, are not by themselves gender transformative (Camilletti et al., 2022.; Peterman et al., 2020). While a cash transfer may protect men’s reputations as providers, complementary interventions may be needed to transform existing gender norms to be more equitable and reflective of the reality of men and women’s contributions. Complementary ‘plus’ interventions could include couples or parenting programming, household or community dialogue, or other programming that explicitly aims to change gender norms around women’s empowerment. For example, Lachman et al. (2021) found that a group-based parenting program added onto a government conditional cash transfer program in the Philippines reduced female caregivers’ experience of IPV. In Rwanda, a home-visiting early childhood development program linked to the social protection system also showed reductions in female caregivers’ experience of IPV at 12-months post intervention (Jensen et al., 2021). Ethiopia’s Productive Safety Net Programme included complementary activities, such as engaging men in nutrition behavior change, that showed promise for improving gender relations and decreasing IPV (Ranganathan et al., 2022). However, qualitative findings from Ethiopia also highlighted tensions related to women’s labor availability to complete cash/food-for-work activities along with household domestic tasks, which need to be considered in the design of future programs.

Our study has several limitations. Our purposeful sample of thirty-five men in two rural districts of northern Ghana may not reflect men’s perceptions of gender and IPV in other regions, or for households that are ineligible for LEAP 1000. In addition, two respondents in one FGD in Yendi district were not fluent in Dagbani and spoke Konkonba, which, although the FGD moderator understood, may have decreased their interactions with the larger group. Finally, our sampling strategy targeted male partners of women participants, but did not include any sampling criteria related to history of IPV to protect our women respondents. This sampling allowed us to capture men’s general perspectives on cash transfer programs, gender and IPV; however, we were not able to deeply probe into experiences and may have missed nuances in how the cash transfer decreased IPV through other mechanisms. Nevertheless, we emphasize the added value of capturing both men and women’s perspectives of cash and IPV – understanding multiple perspectives, and identifying where they converge and diverge provides depth in our understanding of cash and IPV context and pathways.

## 5. Conclusion

This study adds to a growing literature unpacking nuances in the mechanisms through which cash transfer programs affect IPV (Buller et al., 2018). Results confirm the potential of cash plus programming to reduce poverty-related stress among men and women, improve relationship dynamics, and decrease the likelihood of IPV. Financial support provided by cash transfer programs to women provide men with gender-role strain relief and protection from shame from both household and community members, both triggers of IPV. Adding men’s perspectives expands understanding of how cash transfer programs can impact IPV in the context of extreme poverty and hegemonic masculinity, as well as exposes limitations of such programs. Future research including relational measures and data between men and women can help identify cash-plus strategies that are successful in transforming gender norms without placing women at further risk of IPV.

## APPENDIX

**Table:** Key Indicators from Study Districts

District	Yendi Municipal	Garu-Tempane
Capital	Yendi	Garu-Tempane
12010 Population	117,780	130,003
Major ethnic groups	Dagomba, Konkomba	Kusasis, Busangas, Mosis, Bimobas and Mamprusis
Major ethnic language	Dagbani, Lekpakpal	Kusal, Hausa
Average household size	4.5	7.3
Adults with no education	65%	60%
*Marital status*		
Married adult	55%	53.6%
Divorced adults	1.1%	0.8%
Percentage with one or more co-wives*	*Northern region*	*Upper east region*
	38.4%	25.6%
*Intimate partner violence in past* 12 months^†^		
Emotional	62%	
Physical	38%	
Sexual	19%	
Any	68%	
*Religion*		
Islam	67%	41%
Christian	7%	40%
Traditionalist	13%	16%
Other	13%	3%
*Health services (number per district)* Hospital	1	0
Health Center/Clinic	4	19
Community Health and Planning Services (CHPS)	14	43
*Economy (number per district)*		
Markets	4	14
Banks	4	2

Notes:

* among currently married women aged 15–49 years based on the Ghana Statistical service GSS & Ghana Health Service GHS, 2018 Maternal Health Survey DHS. Overall in Ghana, 14 percent of women in this age group reported having one or more co-wives.

† Estimates from the treatment group of the Ghana LEAP 1000 program impact evaluation baseline survey. Source: (Ghana Statistical Service, 2010a, 2010b; Ghana Statistical Service (GSS), Ghana Health Service (GHS), and ICF International, 2018; Kaburi et al., 2017; Ministry of Finance, 2016a, 2016b; Peterman et al., 2022).
